# Ginger extract versus Loratadine in the treatment of allergic rhinitis: a randomized controlled trial

**DOI:** 10.1186/s12906-020-2875-z

**Published:** 2020-04-20

**Authors:** Rodsarin Yamprasert, Waipoj Chanvimalueng, Nichamon Mukkasombut, Arunporn Itharat

**Affiliations:** 1 Department of Applied Thai Traditional Medicine, Faculty of Medicine, Thammasat University, Klongluang, Pathumthani, 12120 Thailand; 2 Department of Otolaryngology, Faculty of Medicine, Thammasat University, Klongluang, Pathumthani, 12120 Thailand; 3 Center of Excellence on Applied Thai Traditional Medicine Research (CEATMR), Faculty of Medicine, Thammasat University, Klongluang, Pathumthani, 12120 Thailand

**Keywords:** Ginger extract, Loratadine, Allergic rhinitis, Quality of life, Clinical trials

## Abstract

**Background:**

Allergic rhinitis (AR) is a non-infectious immune disease and incidents of the disease has continuously increased in Thailand. Ginger, a Thai herb, is used in food and Thai traditional medicine. This study was designed to assess efficacy and safety of ginger extract in comparison with loratadine for AR treatment.

**Methods:**

AR patients were treated with ginger extract 500 mg (*n* = 40) against those treated with loratadine 10 mg (*n* = 40) in a randomized, double-blind, controlled trial for 3 and 6 weeks. The efficacy was evaluated from clinical examinations i.e. total nasal symptom scores (TNSS), cross-sectional area of the nasal cavity with acoustic rhinometry (ARM) and rhinoconjunctivitis quality of life questionnaire (RQLQ). The safety of treatment was measured by blood pressure, blood analysis and history-taking for side effects.

**Results:**

The results showed both ginger extract and loratadine treated groups significantly decreased TNSS scores but there was no significant difference between the two groups. In acoustic rhinometry measurement, the ginger treated group significantly gradually increased the estimated volume of the nasal cavity and decreased distances from the nostril, but the loratadine treated group did not cause a change. Both groups gave significantly improvement in every aspect of the RQLQ at third weeks. The treatment with ginger extract was as safe as loratadine as shown by renal and liver function results obtained from blood analysis. Both treatments had no effect on blood pressure of the patients.

**Conclusions:**

The ginger extract is as good as loratadine in improving nasal symptoms and quality of life in AR patients. However, ginger extract caused less side effects especially, drowsiness, fatigue, dizziness and constipation. Therefore, the ginger extract could be used as alternative treatment for patients with AR.

**Trial registration:**

Registered with ClinicalTrials.gov (Registration number: NCT02576808) on 15 October 2015.

## Background

AR poses a significant global health problem. It is the most common form of non-infectious rhinitis, affecting 10 to 30% of all adults and up to 40% of children. From Epidemiological studies, the worldwide incidents of AR continue to increase. The World Health Organization has estimated that 400 million people in the world are suffering from AR [1]. AR results from specific IgE-mediated allergic reactions in the nasal mucosa and is characterized by a nasal congestion, nasal itching, watery nasal discharge or runny nose, and sneezing [2]. Management of allergic rhinitis has usually focused on suppressing these inflammatory reactions and the main medications are antihistamines, nasal steroids, and leukotriene receptor antagonists [3]. Nowadays, the second-generation non-sedating antihistamines are considered first-line treatment and particularly useful in the treatment of AR. However, anti-histamine has side effects, for example drowsiness, dry mouth, rash or fatigue, etc. [4]. For these reasons it is essential to search for a better-tolerated alternative, especially from herbs.

Ginger (*Zingiber officinale* Roscoe) is widely used as a spice throughout the world. In Thai traditional medicine, it has been used as a part of herbal remedies for treating cold, constipation, sleeplessness and relieving flatulence, etc. [5]. In other traditions such as Indian and Chinese medicine, ginger has been used for several disorders such as asthma, nausea and arthritis [6]. There is evidence to indicate that the ethanolic extract of ginger exhibited the highest anti-allergic activity by inhibited β-hexosaminidase release in rat basophilic leukemia (RBL-2H3) cells. Moreover, 6-shogaol and 6-gingerol is major biomarker of anti-allergic activity [7]. In an *in vivo* study, oral administration of 2% ginger diet decreased the severity of nasal rubbing and sneezing by nasal sensitization of ovalbumin (OVA) and suppressed infiltration of mast cells in nasal mucosa and release of OVA-specific IgE in serum. Furthermore, 6-gingerol (50 μM) could inhibited cytokine production for T cell activation and proliferation, therefore B cell and mast cell could not be activated [8]. In acute and sub-acute toxicity studies, single oral doses of crude ethanolic extract of ginger at 1000, 3000, and 5000 mg/kg body weight did not cause mortality in any animal during the investigation period [9]. In addition to this, ginger extracts have been reported to have a wide range of pharmacological properties and many clinical trials have examined the clinical effectiveness of ginger for conditions such as motion sickness [10, 11], nausea and vomiting [12], osteoarthritis [13–15], and diabetes mellitus [16]. However, there has been no clinical report of ginger extract relieving symptoms in patients with AR.

In this study, we conducted a randomized control trial of ginger extract and loratadine; a commonly-used non-sedating antihistamine to compare the efficacy and safety of these treatments.

## Methods

### Ginger collection and preparation

**Fig. 1:**
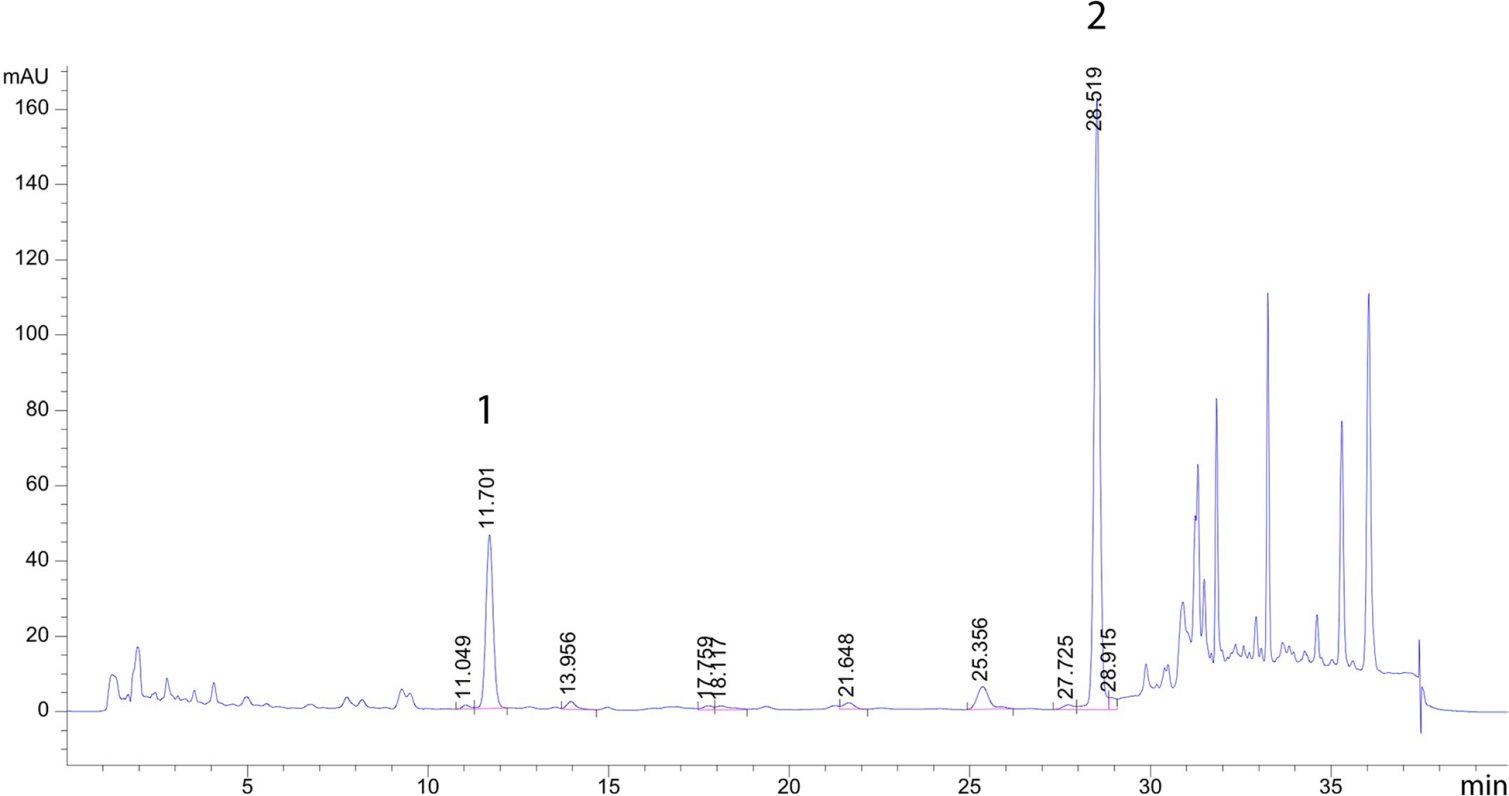
HPLC chromatogram of ginger extract (1 mg/ml). (1) 6-gingerol, (2) 6-shogaol. Mobile phase; water: acetonitrile with gradient elution as follow 0 min, 60:40; 25 min, 50:50; 30 min, 5:95; 35 min, 0:100; 35.10 min, 60:40; Flow rate 1.0 min/ml; UV detector at 227 nm

The fresh rhizomes of ginger were collected in May, 2015 from Ratchaburi province, Thailand. The voucher specimen (BKF 192198) was deposited by Office of the Forest Herbarium, Department of National Parks, Wildlife and Plant Conservation, Bangkok, Thailand and was identified by Mr. Sukid Rueangruea, Forestry Technical Operations Investigators Plant Species official, Bangkok Forest Herbarium, Herbarium Department of National Parks, Wildlife and Plant Conservation, Thailand. The ginger rhizomes were cleaned, steamed by autoclave and dried with hot air oven at 50 °C. The quality standards of ginger rhizomes were applied with the following parameters: contamination testing, loss on drying (moisture content), total ash, acid insoluble ash for inorganic contamination, extractive value and heavy metal content [17]. The dried rhizomes were mechanically powdered and extracted by maceration with 95% ethanol (Liquid: Solid ratio: 1:1) for 3 days and filtered. These were repeated twice, the combined filtrates were concentrated under reduced pressure by a rotary evaporator (Rotavapor R-205, Buchi, Switzerland). Biological quality control of ginger extract was conducted by an anti-allergic assay using the inhibitory effect on β-hexosaminidase in which IC_50_ not more than 30 μg/ml. The high-performance chromatography (HPLC) was also performed to ensure the composition of 6-gingerol and 6-shogaol. HPLC analysis of the study was carried out according to the method of Pattanacharoenchai [18]. Chromatogram of ginger extract and standard compound are shown in Fig.1. From HPLC analysis, the mean contents of 6-gingerol and 6-shogaol in ginger extract were 71.13 and 19.65 mg/g of extract, respectively.

### Drug preparation

The ginger extract was weighed and combined with necessary excipients, and then filled into 500 mg capsules (red-black capsules for the morning meal and white-blue capsules for the evening meal) each containing 125 mg of the ginger extract, produced according to Good Manufacturing Practice (GMP) for Traditional Medicine. Ginger extract capsules were packed in aluminium foil complied with the quality standards of Thai Herbal Pharmacopeia, contamination testing, weight variation and dissolution. Loratadine (Clarityne®) tablets containing 10 mg of micronized loratadine were encapsulated in the same size and color as ginger extract. Lactose monohydrate as a placebo was prepared in a 500 mg capsule.

### Study design

This study was a prospective randomized, double blind, controlled trial (Phase 2), designed to investigate the efficacy and safety of ginger extract compared with loratadine for treating AR patients at Thammasat University Hospital, Pathumthani, Thailand. Before the commencement of the study, the study protocol and informed consent were approved by the Medical Ethics Committee of the Faculty of Medicine, Thammasat University (registry number MTU-EC-TM-4-077/57) and also was registered at ClinicalTrials.gov (NCT02576808).

### Study population and protocol

The sample size determination was calculated from this formula, *N* (each group) = (r + 1)(Z_α/2_ + Z_1-β_)^2^ σ^2^/ rd^2^ [19] where Zα is the normal deviate at a level of significance (Zα is 1.96 for 5%) and Z_1-β_ is the normal deviate at 1-β% power with β% of type II error (0.84 at 80% power. *r* = n1/n2 is the ratio of sample size required for 2 groups, generally it is one for keeping equal sample size for 2 groups. σ and d are the pooled standard deviation and difference of means of 2 groups.

From conducting a pilot study, the minimal detectable difference means (d) of two group as 0.66 scores of total nasal symptom scale (TNSS) and 1.01 is standard deviation (σ).

Thus, the minimum sample size for each group to detect the mean difference between the two means is 36 persons/group. Lastly, considering 10% of drop-out was count out, so forty patients per each treatment group were required for the study.

Eighty patients from the Department of Ear Nose and Throat, Thammasat University Hospital were between 18 and 70 years old were chosen**.** The patients had a clinical history of AR symptoms (itching, nasal congestion, watery nasal discharge or runny nose and sneezing) and were diagnosed by doctor with a moderate AR; minimum TNSS scores of 7 points. Patients could stop taking antihistamine or intranasal steroids for 1 week before trial and did not have history of the following disease: heart disease, kidney disease, liver disease, epilepsy, high blood pressure and severe asthma. Exclusion criteria included patients having fever, taking anti-coagulant, anti-platelet aggregation, erythromycin, clarithromycin**,** ketoconazole, itraconazole and fluconazole**,** experienced serious side effects from loratadine and ginger allergy. Pregnant and lactating women were also excluded.

Informed consent was obtained from the patients who were eligible for the study. The patients were randomly divided into 2 groups (1:1) by using a computer-generated program ensuring no contact with investigators. The patient received a randomized code number sequentially from a secret random list. Treatment assignment was also concealed from all investigators involving in the trial. The masking was opened in medical emergency or if trial successfully accomplished, opened after data analysis.

All patients were instructed about the same appearance of treatment and to take two capsules two times daily for 6 weeks; the experimental group received ginger extract capsules, or the control group, received loratadine. In this study, all patients were followed up at 3rd week and 6th week for evaluating the efficacy, safety, and patient compliance.

### The clinical efficacy evaluation

The efficacy was evaluated by total nasal symptom scores (TNSS) and secondary efficacy variables were measuring the cross-sectional area of the nasal cavity with acoustic rhinometry (ARM) and rhino conjunctivitis quality of life questionnaire (RQLQ).

TNSS score(s), a subjective evaluation as a primary effective tool to measure the intensity symptoms of patients with AR [20], Overall assessment of nose symptoms uses four aspects: runny nose, itchy nose, nasal congestion and sneezing with the score of 4 (0 = no symptoms - 3 = severe symptoms). The total possible score ranged from 0 (no symptoms) to12 (maximum symptom intensity) [3].

ARM is one of the standard diagnostic tools in objective evaluation of nasal patency. ARM can detect minimal cross section area (MCA); narrow points within the nose that may lead to nasal blockage, volume estimates of the nasal cavity (Vol.) and distance from the nostril (Dis.). The reliability of the method is greatest in the anterior nasal cavity, which is the site of the nasal valve [21].

The RQLQ has 28 questions in 7 domains (activity limitation, sleep problems, nose symptoms, eye symptoms, non-nose/eye symptoms, practical problems and emotional function). There are 3 patient-specific questions in the activity domain which furnish patients to choose 3 activities in which they are mostly limited by their rhino conjunctivitis. Patients gave responses to each question on a 7-point scale (0 = not impaired at all - 6 = severely impaired). The overall RQLQ score is the mean of all 28 responses and the individual domain scores are the means of the items in those domains [22].

### The safety evaluation

The safety is measured by using blood analysis, measuring blood pressure and questionnaire. All patients had blood analysis is preformed, three times (before treatment, 3rd week and 6th week). A 10 cc. of blood was taken from each patient in the morning at 7:00 to 9:00 am after 8 h of fasting. The blood specimen were analysed by The Bangkok Pathology-Laboratory including; liver function test and renal function test. All patients were requested to immediately contact the investigator if they noticed any kind of adverse reactions.

### Statistical analysis

All statistical analyses were performed using the standard statistical software. The independent *t*-test or Mann-Withney *U* test was used to compare these mean values between the 2 groups. The repeated measured analysis of variance (ANOVA) or Friedman’s test was used to analyze the changes in the mean values from baseline to 3rd week and 6th week for each group. TNSS score, Total score of RQLQ and ARM values were examined by multivariate regression analyses. Independent variables including treatment with confounders selected demographic and clinical variables (age, gender, body mass index and using steroids). A *p-*value of < 0.05 was considered to indicate statistical significance.

## Results

### Patient characteristics

**Fig. 2:**
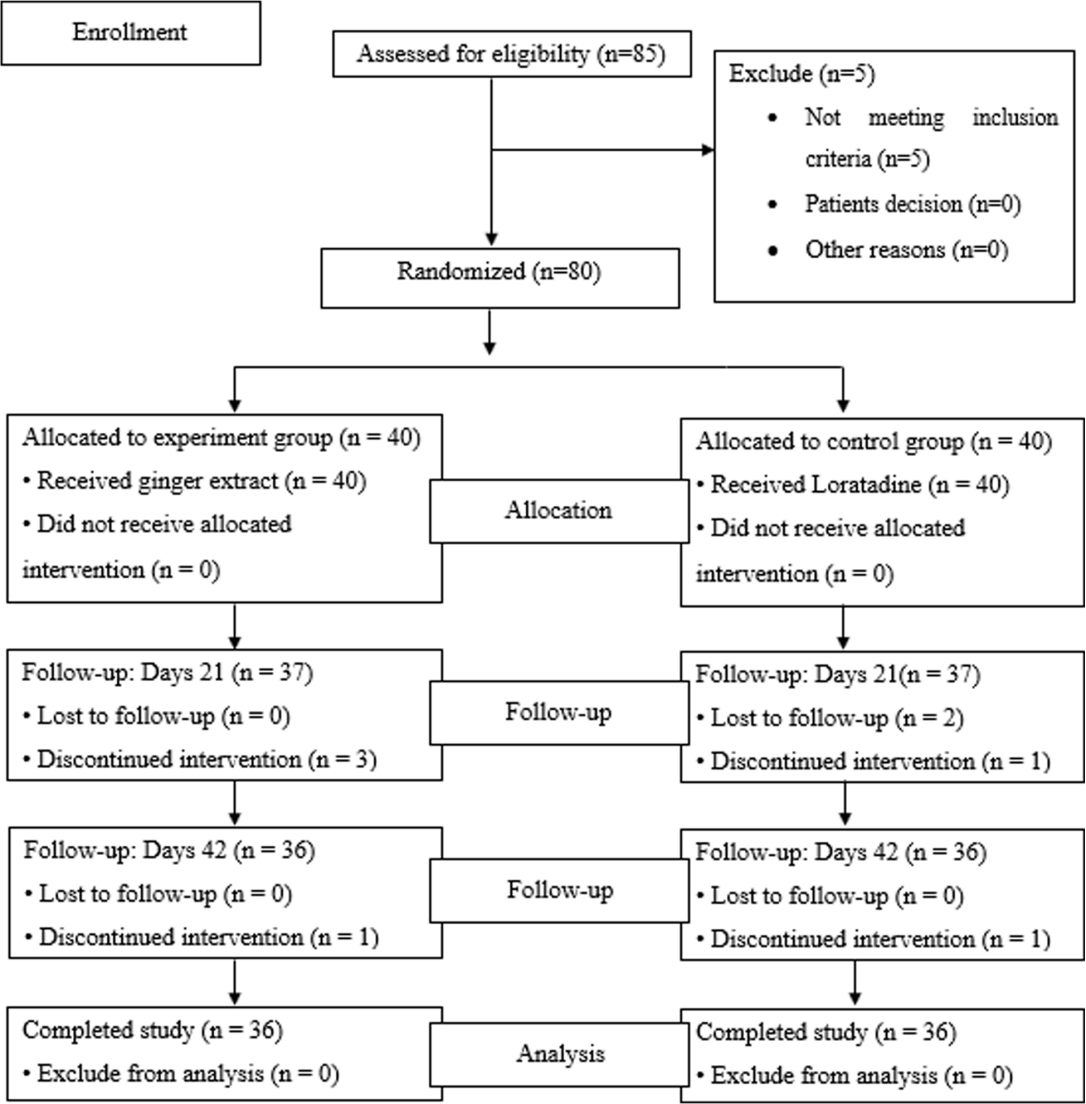
Enrolment and randomization of study subjects

Eighty-five patients were initially screened between October 2016–January 2017 and 5 patients were excluded from the study due to abnormal liver function tests. Thus, 80 patients were randomized into 2 groups (40 patients in each group). There was no significant difference between the two groups in age, gender, underlying diseases of AR and laboratory data (Table 1). After the end of study, 72 patients (90%) completed the study (36 patients in the ginger extract treated group and 36 patients in the loratadine treated group). Eight patients were withdrawn during the study due to failing the follow-up (six patients dropped out at the first follow up and two patients dropped out at the second follow up)**.** The reasons for withdrawn as follow: in ginger extract treated group, two patients used other anti-histamine, one patient had food poisoning and one patient experienced nausea and dizziness. In loratadine treated group**,** one patient was unsatisfied with the efficacy of loratadine**,** one patient had Hepatitis A and two patients left the trial (Fig. 2).

**Table 1:** Baseline characteristics of patients

Data	Ginger extract (*n* = 40)	Loratadine (*n* = 40)	*p*-value*
Female, number (%)	28 (70)	30 (75)	0.617^c^
Age; yrs., mean (SD)	35.42 (12.73)	30.75 (9.72)	0.069^a^
BMI; Kg/m^2^, mean (SD)	21.92 (3.34)	21.87 (2.99)	0.946 ^b^
Exercise history, number (%)	18 (45)	25 (62.5)	0.116^c^
Total TNSS score, mean (SD)	7.48 (1.96)	7.37 (2.32)	0.835^t^
MCA (cm^2^), mean (SD)			
Right MCA	0.32 (0.14)	0.31(0.14)	0.661^a^
Left MCA	0.34 (0.16)	0.31 (0.18)	0.378^a^
Volumes (cm^3^) of nasal cavity			
Right Vol	3.83 (0.98)	3.62 (1.04)	0.365^a^
Left Vol	3.95 (1.11)	3.53 (1.16)	0.103^a^
Distance (cm)			
Right Dis	2.11 (0.31)	2.22 (0.25)	0.091^a^
Left Dis	2.11 (0.28)	2.27 (0.43)	0.068^a^
Total RQLQ score, mean (SD)	2.98 (0.99)	3.12 (1.12)	0.546^a^
Laboratory data, mean (SD)			
Blood pressure			
Systolic (mm. Hg.)	117.05 (9.60)	115.28 (13.16)	0.631 ^b^
Diastolic (mm. Hg.)	75.95 (9.49)	72.10 (10.15)	0.059 ^b^
Renal function tests			
BUN (mg/dL)	11.81 (3.47)	11.50 (3.59)	0.698^a^
Creatinine (mg/dL)	0.74 (0.18)	0.74 (0.18)	0.956^a^
Liver function tests			
AST (U/L)	21.13 (6.71)	20.85 (8.73)	0.875^a^
ALT (U/L)	28.38 (11.96)	27.05 (13.35)	0.641^a^
ALP (U/L)	60.93 (13.68)	65.88 (21.93)	0.229^a^
Eosinophil	3.65 (2.52)	3.92 (2.45)	0.829 ^b^
Basophil	0.51 (0.43)	0.40 (0.38)	0.308 ^b^

*statistical analysis: ^a^ independent two-sample Student’s t-test, ^b^ Mann Whitney U Test and ^c^chi-square test

### The clinical efficacy evaluation

The results showed that the ginger extract and the loratadine treated groups significantly decreased the TNSS scores with no statistically significant difference between the two treated groups. The four main symptoms were separately assessed i.e. itching, runny nose, nasal congestion and sneezing the first 3 symptoms decreases in the third week. The sneezing symptom, in the ginger treated groups showed significant reduction in week 6 but loratadine treatment could reduce sneezing in 3 weeks (Table 2).

**Table 2:** The score of total nasal symptoms scores of ginger extract and loratadine

Data^a^	Follow-up	Treatment^b^		*p*-value***
		Ginger extract	Loratadine	
Total TNSS score	Week 0	7.48 (1.96)	7.38 (2.32)	0.835
	Week 3	4.30 (2.47) †††	4.33 (2.57) †††	0.989
	Week 6	3.42 (2.80) †††	4.11 (2.56) †††	0.276
runny nose	Week 0	2.00 (0.82)	2.00 (1.04)	1.000
	Week 3	1.19 (0.82) †††	1.28 (0.91) ††	0.812
	Week 6	0.89 (0.78) †††	1.14 (0.96) †††	0.231
itchy nose	Week 0	1.65 (0.86)	1.60 (0.87)	0.797
	Week 3	0.89 (0.88) †††	0.81 (0.79) †††	0.796
	Week 6	0.81 (0.88) †††	0.86 (0.87) †††	0.789
nasal congestion	Week 0	2.32 (0.62)	2.15 (0.77)	0.265
	Week 3	1.19 (0.81) †††	1.31 (0.95) †††	0.718
	Week 6	1.00 (0.16) †††	1.28 (0.88) ††	0.204
sneezing	Week 0	1.50 (0.99)	1.62 (0.93)	0.561
	Week 3	1.03 (0.93)	0.94 (0.71) †††	0.681
	Week 6	0.72 (0.81) †	0.83 (0.77) †††	0.555

^a^Data represent mean (SD), ^b^Statistical analysis: repeated measured ANOVA, †Significant difference from day 0 within group (*p* < 0.05), ††significant difference from day 0 within group (*p* < 0.01), and†††significant difference from day 0 within group (*p* < 0.001)

*** Statistical analysis: Independent two-sample Student’s t-test

In ARM performed, the ginger extract treated group gradually increased in minimal cross section area at week 3 but not statistically significant. The volume estimates of nasal cavity were significantly improved at week 6. On the other hand, loratadine treated group did not show improvement. When comparing the differences between the two treated groups, the result showed that there was significant difference in the volume at week 6 (Table 3).

**Table 3:** The acoustic rhinometry parameter of ginger extract and loratadine

Data^a^	Follow-up	Treatment^b^		*p*-value***
		Ginger extract	Loratadine	
Minimal cross section area of right nose (cm^2^)	Week 0	0.32 (0.14)	0.31 (0.14)	0.661
	Week 3	0.34 (0.15)	0.32 (0.14)	0.771
	Week 6	0.37 (0.15)	0.32 (0.14)	0.120
Minimal cross section area of left nose (cm^2^)	Week 0	0.34 (0.16)	0.31 (0.18)	0.378
	Week 3	0.36 (0.14)	0.31 (0.13)	0.164
	Week 6	0.35 (0.10)	0.31 (0.13)	0.160
Volume estimates of the right nasal cavity (cm^3^)	Week 0	3.83 (0.98)	3.62 (1.04)	0.365
	Week 3	3.95 (1.18)	3.84 (1.21)	0.685
	Week 6	4.38 (1.42) †	3.63 (1.20)	0.018*
Volume estimates of the left nasal cavity (cm^3^)	Week 0	3.95 (1.11)	3.53 (1.16)	0.103
	Week 3	4.28 (1.28)	3.65 (0.87)	0.014*
	Week 6	4.25 (0.99) †	3.67 (1.15)	0.027*
Distance from the nostril of right nose (cm)	Week 0	2.11 (0.31)	2.22 (0.25)	0.091
	Week 3	2.15 (0.30)	2.16 (0.30)	0.828
	Week 6	2.19 (0.21)	2.16 (0.32)	0.607
Distance from the nostril of left nose (cm)	Week 0	2.11 (0.28)	2.27 (0.43)	0.068
	Week 3	2.11 (0.29)	2.20 (0.32)	0.245
	Week 6	2.07 (0.35)	2.26 (0.29)	0.011*

^a^Data represent mean (SD), ^b^Statistical analysis: repeated measured ANOVA, †Significant difference from day 0 within group (*p* < 0.05), ††significant difference from day 0 within group (*p* < 0.01), and†††significant difference from day 0 within group (*p* < 0.001)

*** Statistical analysis: Independent two-sample Student’s t-test

After treatment for 3 weeks*,* the quality of life of both treated groups significantly improved in every aspect scores. (Table 4).

**Table 4:** The score of quality of life of ginger extract and loratadine

Data^a^	Follow-up	Treatment		*p*-value***
		Ginger extract	Loratadine	
Total RQLQ score	Week 0	2.98 (0.99)	3.12 (1.12)	0.547
	Week 3	1.88 (0.96) †††	1.92 (1.17) †††	0.881
	Week 6	1.34 (0.95) †††	1.44 (1.06) †††	0.660
Activity limitation	Week 0	3.95 (1.08)	4.32 (0.99)	0.119
	Week 3	2.64 (1.31) †††	2.98 (1.41) †††	0.279
	Week 6	1.75 (1.27) †††	2.15 (1.36) †††	0.211
Sleep problems	Week 0	3.00 (1.46)	2.93 (1.58)	0.827
	Week 3	1.87 (1.32) †††	1.50 (1.22) †††	0.209
	Week 6	1.13 (1.23) †††	1.18 (1.24) †††	0.861
Non-nose/eye symptoms	Week 0	2.82 (1.28)	2.96 (1.44)	0.653
	Week 3	1.65 (1.01) †††	1.92 (1.40) ††	0.339
	Week 6	1.30 (1.13) †††	1.37 (1.19) †††	0.819
Practical problems	Week 0	3.07 (1.52)	3.50 (1.55)	0.210
	Week 3	2.04 (1.34) ††	2.17 (1.50) †††	0.713
	Week 6	1.57 (1.35) †††	1.59 (1.22) †††	0.946
Nose symptoms	Week 0	3.48 (1.20)	3.92 (1.38)	0.129
	Week 3	2.28 (1.30) †††	2.33 (1.33) ††	0.864
	Week 6	1.67 (1.36) †††	1.96 (1.31) †††	0.379
Eye symptoms	Week 0	2.39 (1.44)	2.28 (1.81)	0.772
	Week 3	1.47 (1.44) †††	1.28 (1.50) †††	0.578
	Week 6	1.05 (1.16) †††	1.00 (1.28) †††	0.864
Emotion	Week 0	2.32 (1.53)	2.10 (1.54)	0.537
	Week 3	1.46 (1.22) †††	1.31(1.33) †††	0.627
	Week 6	0.94 (0.95) †††	0.96 (1.06) †††	0.948

^a^Data represent mean (SD), **Statistical analysis: repeated measured ANOVA, †Significant difference from day 0 within group (*p* < 0.05), ††significant difference from day 0 within group (*p* < 0.01), and†††significant difference from day 0 within group (*p* < 0.001)

*** Statistical analysis: Independent two-sample Student’s t-test

After adjusting for possible differences in clinical characteristics between the treatment groups, the results showed that the TNSS scores of the ginger extract treated group consistently decreased at week 3 and 6 and were better than loratadine group, (0.666 and 0.574 scores, respectively). As for ARM value, the ginger extract treated group significantly increased the volume of left nose with 0.094 cm^3^ (*p* = 0.02) and decreased distance of left nose with 0.023 cm (*p* < 0.01). In contrast, loratadine treated group did not show significant improvement. In total score of RQLQ, the ginger extract group showed reduced score with 0.283 points but no significant difference from loratadine group with 0.266 points (Table 5).

**Table 5:** Clinical efficacy change score by multivariate regression analyses parameter estimates

Data	Treatment	Mean ± SD	95% Conf.Interval		*p*-value
			lower	upper	
TNSS score	Loratadine	−0.574 (0.709)	−0.416	−0.732	0. 343
	Ginger extract	−0.666 (0.649)	− 0.522	− 0.811	
Minimal cross section area of right nose (cm^2^)	Loratadine	0.004 (0.032)	−0.003	0.011	0.551
	Ginger extract	0.007 (0.037)	−0.001	0.016	
Minimal cross section area of left nose (cm^2^)	Loratadine	−0.001 (0.033)	−0.008	0.006	0.2357
	Ginger extract	0.005 (0.033)	−0.002	0.012	
Volume estimates of the right nasal cavity (cm^3^)	Loratadine	0.011 (0.285)	−0.052	0.075	0.106
	Ginger extract	0.086 (0.326)	0.014	0.159	
Volume estimates of the left nasal cavity (cm^3^)	Loratadine	−0.006 (0.265)	−0.065	0.053	0.02*
	Ginger extract	0.094 (0.288)	0.030	0.158	
Distance from the nostril of right nose (cm)	Loratadine	−0.003 (0. 076)	−0.020	0.014	0.402
	Ginger extract	0.006 (0. 066)	−0.008	0.021	
Distance from the nostril of left nose (cm)	Loratadine	0.010 (0. 079)	−0.008	0.027	0.008*
	Ginger extract	−0.023 (0. 097)	−0.045	− 0.002	
Total RQLQ score	Loratadine	−0.266 (0.299)	−0.199	− 0.332	0.701
	Ginger extract	−0.283 (0.276)	−0.222	− 0.345	

Statistical analysis: multivariate regression

### The safety evaluation

The side effects having highest occurrence in ginger extract treated group were eructation (72.22%), dry mouth (11.11%) and throat (11.11%). In loratadine group, drowsiness was the most common event (25%) and other side effects, for example dry throat, eructation, dry mouth (19.44, 16.67 and 13.89%, respectively) (Table 6). In both groups, the systolic and diastolic blood pressure measurements were not significantly different from baseline and also not significantly different between treated groups (Table 7). All patients were examined for blood urine nitrogen (BUN) and creatinine for renal function tests and aspartate transaminase (AST), alanine aminotransferase (ALT), and alkaline phosphatase (ALP) for liver function tests at third and sixth weeks. The renal function was similar in both groups when compared with their baseline values. For liver function tests, in both treated groups were not significantly different AST and ALT level from baseline. Moreover, the ginger extract treated group slightly decreased ALP levels at week 6 while the loratadine treated group showed an increased ALP level which was significantly difference from ginger extract treated group.

**Table 6:** Side effects of Ginger extract and Loratadine

Side effect	Ginger extract (***n*** = 36) Number (%)	Loratadine (***n*** = 36) Number (%)
eructation	26 (72.22)	6 (16.67)
drowsiness	1 (2.78)	9 (25)
dry mouth	4 (11.11)	5 (13.89)
dry throat	4 (11.11)	7 (19.44)
keen nose	0	2 (5.56)
fatigue	1 (2.77)	4 (11.11)
dizziness	1 (2.77)	3 (8.33)
constipation	0	3 (8.33)

**Table 7:** Blood pressure, renal functions, and liver functions in safety issue

Data^a^	Treatment	Week 0	Week 3	Week 6	*p*-value**
**Blood pressure**					
Systolic blood pressure	Ginger extract	117.05 (9.60)	113.76 (9.73)	111.54 (17.91)	0.767
(Normal ≤140 mm.Hg.)	Loratadine	115.28 (13.16)	114.37 (12.44)	114.25 (14.86)	
Diastolic blood pressure	Ginger extract	75.95 (9.48)	73.73 (9.99)	74.58 (12.65)	0.112
(Normal ≤90 mm.Hg.)	Loratadine	72.10 (10.15)	70.08 (11.74)	71.19 (11.88)	
**Renal functions**					0.729
Blood urea nitrogen; BUN (mg/dL)	Ginger extract	11.81(3.47)	11.26 (2.48)	10.77 (3.32)	
(ref. range = 7.0–18.0)	Loratadine	11.50 (3.59)	11.32 (2.71)	10.61 (2.26)	
Creatinine (mg/dL)	Ginger extract	0.74 (0.18)	0.76 (0.19)	0.74 (0.23)	0.826
(ref. range = 0.7–1.3)	Loratadine	0.74 (0.18)	0.75 (0.18)	0.75 (0.16)	
**Liver functions**					
AST (U/L) (ref. range = 15–37)	Ginger extract	21.13 (6.71)	19.81 (5.18)	20.19 (6.92)	0.871
	Loratadine	20.85 (8.73)	24.38 (14.66)	20.56 (5.95)	
ALT (U/L) (ref. range = 30–65)	Ginger extract	28.38 (11.96)	27.51 (12.92)	26.78 (11.39)	0.586
	Loratadine	27.05 (13.35)	29.51 (20.52)	27.08 (12.28)	
ALP (U/L) (ref. range = 46–116)	Ginger extract	60.93 (13.68)	62.30 (16.73)	58.11(17.66)	0.118
	Loratadine	65.88 (21.93)	67.89 (25.00)	68.72 (25.04)	

^a^Data represent mean (SD), **Statistical analysis: repeated measured ANOVA, †Significant difference from day 0 within group (*p* < 0.05), ††significant difference from day 0 within group (*p* < 0.01), and†††significant difference from day 0 within group (*p* < 0.001)

## Discussion

Allergic inflammation process is divided into two phases as follows; sensitization phase which is process of IgE production after exposure to the allergen and clinical phase where many symptoms appear during exposure to allergens. The clinical phase is divided into an early phase response, which involves degranulation of mast cells such as histamine, leukotriene C4 (LTC4), prostaglandin D2 (PGD2), release cytokines such as interleukin (IL)- 3, IL-4, IL-5, and IL-13 [2] and tumor necrosis factor-alpha (TNF-a) [23]. In late-phase response, which is associated with an increase in inflammatory cells in the nasal mucosa and increased secretion of cytokines results in recurrent symptoms. AR is an allergic inflammatory disease of the nasal airway causing chronic symptoms that continuously fluctuate in severity over time, discomfort and a decrease in quality of life. For this reason, early symptomatic treatment through inflammation control is important. Because of the chronic nature of allergic inflammation, some patients are reluctant to take long-term medication and so turn to unverified alternative medications.

Ginger is one of the most widely consumed spices worldwide. It has a long history using as herbal medicine to treat a variety of ailments. Many trials examined the clinical effectiveness of ginger for conditions such as osteoarthritis, nausea and vomiting, and flatulence or indigestion.

Management of AR has usually focused on suppressing these inflammatory reactions [3]. Therefore, ginger has a tendency to target the symptoms of AR by anti-allergic and anti-inflammatory mechanisms. This is confirmed by the researchers who found that the ethanolic extract of ginger inhibited allergic reactions in rat basophilic leukemia (RBL-2H3) cells, with an IC_50_ value of 12.93 ± 1.28 μg/ml. Moreover, 6-shogaol and 6-gingerol, the major compounds in ginger extract, exhibited the highest anti-allergic activity at IC_50_ value of 0.28 ± 0.11 mg/ml (1.01 μM) and 18.30 ± 3.38 mg/ml (62.16 μM), respectively [7]. Kawamoto and team studied the anti-allergic effects of ginger and 6-gingerol by using a mouse allergy model. The result presented that 2% dietary ginger reduced the severity of nasal rubbing and sneezing by nasal sensitization of OVA and suppressed infiltration of mast cells in nasal mucosa and secretion of OVA specific IgE in serum. After spleen cells were induced with OVA, 6-Gingerol (50 μM) inhibited the expression Th2 cytokine (IL-4, IL-10 and IL-13) and Th1cytokine (IFN-*γ*) [8].

There are also studies on anti-inflammatory ability which found that ginger has highest anti-inflammatory activity. Ginger extract showed strong inhibitory effect of the release of IL-1b in human peripheral blood mononuclear cells (PBMCs) [24], COX-1 and COX-2 [25]. In previous study, active ingredients, 6-shogaol and 6-gingerol, presented the most potent to reduce TNF-a release [7].

Dose of drugs were followed from Reference Dose (RfD). A reference dose is the United States Environmental Protection Agency’s maximum acceptable oral dose of a toxic substance. RfD is obtained from probabilistic multiplication of NOAEL (No-Observed-Adverse-Effect-Level) value which is tested for acute toxicity, sub-chronic toxicity and chronic toxicity in laboratory animals, and has to be safe and with no undesirable effects. The previous study has shown that ethanolic extract of ginger at 5000 mg/kg did not toxicity in both acute and sub-acute toxicity [9]. Therefore, the calculation maximum dose with no adverse effects of the ginger extract is 3 g or 3000 mg per day. In another clinical study of 261 osteoarthritis patients, 255 mg of ginger extract twice a day for 6 months, can significantly relieve pain better than placebo although patients receiving ginger extract had unpleasant gastrointestinal sensations which were not a serious event [13]. Thus, this study used 500 mg of ginger extract per day per volunteer.

These results urge the conclusion that ginger extract is an excellent anti-allergic and anti-inflammatory agent and it is consistent with this study that taking ginger extract continuously for 6 weeks can relieve the symptoms of allergic rhinitis and improve the quality of life for patients. In addition, there were volunteers who consumed ginger extract to help relieve flatulence and improved defecation at 50 and 23%, respectively.

The TNSS is a widely accepted and reliable tool to assess the efficacy of a drug for treating AR, and the decrease of the score indicate that an overall clinical improvement in the condition. This study showed that AR patients treating with ginger extract could reduce total TNSS and four main symptoms i.e. itching, runny nose, nasal congestion and sneezing.

ARM is used to objectively measure the minimal cross-sectional area and volumes of nasal cavities in various depths when measured from the front into the nostril, by analysing reflections of a sound pulse introduced via the nostrils. The technique is a rapid, reproducible, painless, non-invasive procedure that requires little cooperation of the patients and has been applied to both children and adults. This study shows that the ginger extract group had significantly increased volume and decreased distant of left nose, it means improved nasal congestion. Nasal congestion is related with acute allergic inflammation and chronic inflammation of mast cells. The second-generation non-sedating antihistamines, which are generally effective on suppress histamine-mediated symptoms such as sneezing and nasal discharge, are generally not effective in relieving symptoms of nasal congestion; a phenomenon driven by a number of vasoactive mediators in addition to histamine on mast cells [26]. Therefore, anti-histamines are often prescribed in combination with decongestants, which perform to constrict the blood vessels in the mucous membranes and thus diminish nasal congestion. Therefore, ginger extract may be used to treat AR patients either as single drug or in combination with loratadine in case severe sneezing.

AR has been associated with significant impairments in quality of life, sleep and work performance. Assessment of quality of life has now become a standard of allergy clinical trials. Ginger extract reduced RQLQ scores in every aspect which the results represented ginger could improve their quality of life.

The result of blood analysis did not shown any toxicity, therefore we suggest that ginger extract is safe and can be used to treat AR patients.

This is the first research report on treatment of AR patients with ginger extract and its comparison with loratadine. The limitation of this study was short-term and small-scale study. Future studies long-term period and large-scale are needed to completely evaluate the efficacy and safety of ginger extract.

## Conclusion

This study showed that ginger extract could reduce AR symptoms and is safe to use with very mild GI side effect such as eructation. Ginger extract is better than loratadine in causing less drowsiness, fatigue, dizziness and constipation.

## Data Availability

Dataset of this manuscript has not been deposited in any repository. All dataset and materials are available from the corresponding author upon reasonable request.

## Abbreviations

ALP: Alkaline phosphatase
ALT: Alanine aminotransferase
AR: Allergic rhinitis
ARM: Acoustic rhinometry
AST: Aspartate transaminase
BUN: Blood urea nitrogen
Dis: Distance from the nostril
GMP: Good Manufacturing Practice
HPLC: High-performance chromatography
IL: Interleukin
MCA: Minimal cross section area
PGD2: Prostaglandin D2
RBL-2H3: Rat basophilic leukemia
RQLQ: Rhinoconjunctivitis quality of life questionnaire
TNF-α: Tumor necrosis factor-alpha
TNSS: Total nasal symptom scores
Vol: Volume estimates of the nasal cavity
